# The Synergistic Effect of Intradialytic Concurrent Training and Melatonin Supplementation on Oxidative Stress and Inflammation in Hemodialysis Patients: A Double-Blind Randomized Controlled Trial

**DOI:** 10.3390/antiox13111290

**Published:** 2024-10-25

**Authors:** Houssem Marzougui, Imen Ben Dhia, Ines Mezghani, Rami Maaloul, Salma Toumi, Khawla Kammoun, Mohamed Nejib Chaabouni, Fatma Ayadi, Mohamed Ben Hmida, Mouna Turki, Omar Hammouda

**Affiliations:** 1 Research Laboratory Molecular Bases of Human Pathology LR19ES13, Faculty of Medicine, University of Sfax, Sfax 3029, Tunisia; houssemmg1994@gmail.com (H.M.); mezghani.ines916@gmail.com (I.M.); rami.maaloulll@gmail.com (R.M.); ayadi_fatma@medecinesfax.org (F.A.); mouna.turki@gmail.com (M.T.); 2 High Institute of Sport and Physical Education of Sfax, University of Sfax, Sfax 3000, Tunisia; b.dimene@yahoo.com; 3 Research Laboratory of Evaluation and Management of Musculoskeletal System Pathologies LR20ES09, Faculty of Medicine, University of Sfax, Sfax 3029, Tunisia; 4 Biochemistry Laboratory, CHU Habib Bourguiba, University of Sfax, Sfax 3029, Tunisia; 5 Nephrology Department, CHU Hedi Chaker, University of Sfax, Sfax 3029, Tunisia; toumisalma@rocketmail.com (S.T.); khawlakammoun2002@gmail.com (K.K.); nejib.cnh@planet.tn (M.N.C.); mohamed.benhmida.148@gmail.com (M.B.H.); 6 Research Laboratory of Renal Pathology LR19ES11, Faculty of Medicine, University of Sfax, Sfax 3029, Tunisia; 7 Interdisciplinary Laboratory in Neurosciences, Physiology and Psychology: Physical Activity, Health and Learning (LINP2), UFR STAPS, Paris Nanterre University, 92001 Nanterre, France

**Keywords:** intradialytic exercise, melatonin, end-stage renal disease, oxidative stress, antioxidant, inflammation

## Abstract

Background: This study aimed to investigate the effects of intradialytic concurrent (resistance–endurance) training combined with melatonin (MEL) supplementation on oxidative stress, inflammation, and cellular damage in hemodialysis (HD) patients. Methods: Thirty-two HD patients were randomly assigned to three groups: Exercise (EX)-MEL, EX-Placebo (PLA), and Control (C)-PLA. Participants in the EX-MEL and EX-PLA groups underwent 12 weeks of concurrent training. Before nocturnal sleep, they ingested either 3 mg of MEL (EX-MEL) or a placebo (EX-PLA and C-PLA). Blood samples were collected at baseline and after 12 weeks of intervention to assess lipid peroxidation [malondialdehyde (MDA)], antioxidant biomarkers [ferric-reducing antioxidant power (FRAP), reduced glutathione (GSH), total thiol (THIOL)], total bilirubin (TBIL), uric acid (UA), biomarkers of muscle and liver damage [aspartate aminotransferase (ASAT), alanine aminotransferase (ALAT), creatine kinase (CK), lactate dehydrogenase (LDH), and Gamma-glutamyltransferase (Gamma-GT)], and inflammation [C-reactive protein (CRP)]. Results: EX-MEL demonstrated a decrease in MDA (*p* < 0.05) and CRP (*p* < 0.05), and an increase in FRAP (*p* < 0.05) pre- and post-training. Both EX-MEL and EX-PLA showed an increase in GSH (*p* < 0.001, and *p* < 0.05, respectively) and THIOL (*p* < 0.01, and *p* < 0.05, respectively) pre- and post-training. No significant changes were observed in TBIL, UA, ASAT, ALAT, CK, LDH, or Gamma-GT pre- and post-training across all groups. Conclusion: Concurrent training combined with MEL supplementation enhances oxidant–antioxidant balance and reduces inflammation in HD patients more effectively than intradialytic concurrent training alone.

## 1. Introduction

Hemodialysis (HD) is a critical treatment for patients with end-stage renal disease, but it is often accompanied by severe complications such as increased oxidative stress and inflammation, which can lead to cellular damage and increase the rates of morbidity and mortality in this population [1]. Oxidative stress results from an imbalance between the production of reactive oxygen species (ROS) and antioxidant defenses, leading to damage to lipids, proteins, and DNA [2]. Inflammation, commonly assessed by biomarkers such as C-reactive protein (CRP), is a prevalent issue among HD patients [3]. For instance, increased levels of ROS and pro-inflammatory cytokines have been associated with cardiovascular complications and a poor prognosis in this population [4]. Addressing these issues is crucial for improving patient outcomes and quality of life.

Physical exercise, particularly programs integrating both resistance and endurance training components during HD, known as concurrent training, has emerged as a promising strategy offering significant health benefits for HD patients [5]. These exercise regimens have been associated with improvements in functional capacity, cardiovascular health, and overall quality of life [6,7]. However, despite these benefits, there remains a notable gap in research regarding the specific effects of intradialytic concurrent training (ICT) on oxidative stress and inflammation in this population. Evidence suggests that exercise can reduce oxidative stress and inflammatory markers in the general population, but the impact on HD patients remains underexplored [8,9]. Conducting exercise during dialysis sessions presents an opportunity to potentially alleviate some of the adverse effects associated with HD.

Melatonin (MEL), a hormone primarily known for regulating sleep–wake cycles, also possesses significant antioxidant and anti-inflammatory properties [10]. MEL supplementation could potentially amplify the benefits of exercise by further reducing oxidative stress and inflammation [11,12]. Despite the theoretical benefits, there has been limited research on the combined effects of ICT and MEL supplementation in HD patients [5].

This study aims to explore the synergistic effects of ICT training and MEL supplementation on oxidative stress, inflammation, and cellular damage in HD patients. We hypothesized that this strategy would provide valuable insights into new therapeutic strategies to improve health outcomes and potentially attenuate oxidative stress and inflammation in HD patients.

## 2. Materials and Methods

### 2.1. Sample Size

The sample size for this study was calculated a priori using Beck’s recommended methods [13] and the G*Power program (version 3.1.9.7, Heinrich-Heine University Düsseldorf, Düsseldorf, Germany) [14] to determine the minimum required sample size. The alpha value (α) was set at 0.05, and the power (1-β error probability) was set at 0.95. Following discussions between the authors, the effect size was estimated to be 0.8. To achieve the desired power and minimize the risk of a type II statistical error, data from at least 21 participants were anticipated to be sufficient.

### 2.2. Participants

**Figure 1:**
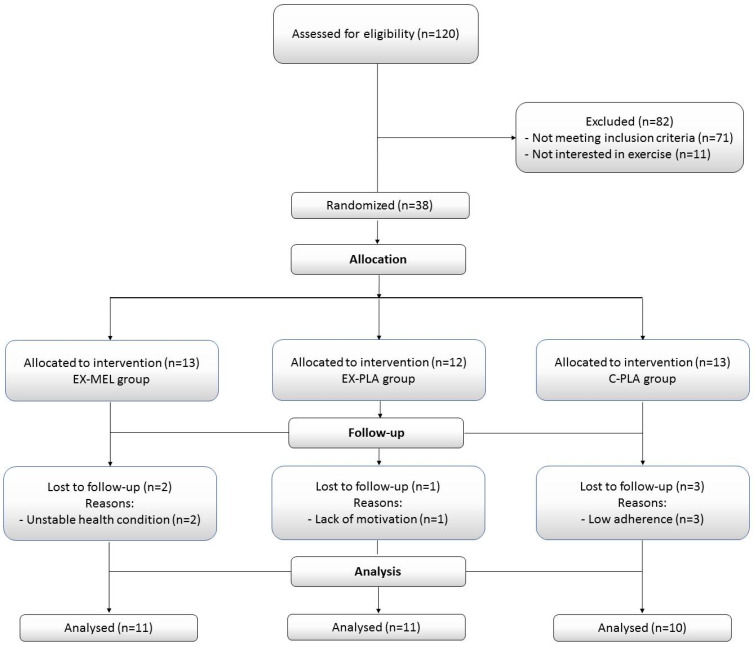
Flowchart of study participants.

Thirty-two participants (average age 49.21 ± 10.56 years) undergoing HD (a four-hour session, three times per week) for at least six months were recruited from the HD unit of the local university hospital, and all of them completed the study protocol. The recruitment period began on 1 June 2020, with the final follow-up completed on 30 September 2020. Figure 1 presents a flowchart detailing the participants’ progress through the study phases, while Table 1 displays the participants’ baseline characteristics.

All participants had an arteriovenous fistula for dialysis access. Participants were excluded from the study if they met any of the following criteria: (i) recent participation in an exercise program within the last six months; (ii) serious conditions necessitating hospitalization or hindering exercise, or severe cardiovascular diseases; (iii) active infectious or inflammatory disease; (iv) orthopedic issues; (v) severe psychological or neurological disorders; (vi) use of antioxidant supplements (i.e., vitamin E, statins, or any other medication with antioxidant properties); and (vii) the use of psychoactive drugs. Participants were randomly assigned to one of three groups: Exercise (EX)-MEL (n = 11), EX-Placebo (PLA) (n = 11), and Control (C)-PLA (n = 10). The dropout rate was set at 15–20% of the total training sessions. All participants received detailed information about the experimental procedures and they provided written informed consent to participate. The study was approved by the South Institutional Human Research Ethics Committee in Sfax, Tunisia (N°0059/2017) and conducted in accordance with the ethical principles of the Declaration of Helsinki (2013) [15]. Additionally, the study is registered with the Pan African Clinical Trials Registry under the registration number PACTR202005770507528.

### 2.3. Experimental Design

This study was a randomized, double-blind, placebo-controlled clinical trial. Participants in the EX-MEL and EX-PLA groups participated in ICT sessions three times per week for 12 weeks. Each evening, 30 min before sleep, the EX-MEL group took 3 mg of MEL (Jamieson Laboratories, Toronto, ON, Canada), while the EX-PLA and C-PLA groups received a placebo (Galpharma Laboratories, Sfax, Tunisia). The 3 mg MEL dose was chosen based on recommendations for HD patients by Koch et al. [16] and Russcher et al. [17], with no significant side effects reported at this dosage. Participants in the EX-MEL and EX-PLA groups were familiarized with the lower-limb cycle ergometer, resistance exercises (including knee extension, hip abduction, and flexion), and the Rating of Perceived Exertion (RPE) scale to minimize learning effects. Importantly, while participants in the C-PLA group were aware that they were not engaging in physical exercise, they were blinded to whether they received MEL or a PLA. Additionally, participants in the EX-MEL and EX-PLA groups were also blinded regarding their supplementation status, ensuring that all groups minimized potential biases related to treatment expectations. Additionally, the height and body weight of the participants were measured using a semi-analytical balance scale, and their body mass index (BMI) was calculated as BMI = weight (kg)/height (m)^2^. Before and after the 12-week intervention period, blood samples were taken from all participants to assess lipid peroxidation [malondialdehyde (MDA)], antioxidant biomarkers [ferric-reducing antioxidant power (FRAP), reduced glutathione (GSH), total thiol (THIOL)], total bilirubin (TBIL), uric acid (UA), biomarkers of muscle and liver damage [aspartate aminotransferase (ASAT), alanine aminotransferase (ALAT), creatine kinase (CK), lactate dehydrogenase (LDH), and Gamma-glutamyltransferase (Gamma-GT)], and CRP. This study followed the CONSORT guidelines for randomized controlled trials. Details regarding adherence can be found in the Supplementary Materials (see CONSORT checklist).

### 2.4. Concurrent Training Program

During the first 2 h of HD, participants in the EX-MEL and EX-PLA performed an ICT program in the HD unit under the supervision of physicians. Each ICT session involved endurance and resistance exercises. The endurance exercise began with a 5-min warm-up, followed by 15 min of cycling on a lower-limb cycle ergometer (Everfit Welly-M, Pozzolo Formigaro, Alessandria, Italy) placed in front of each participant. The exercise duration was gradually increased, adding 5 min at the end of the first week and 10 min at the end of the second week to reach 30 min within the first two weeks. Subsequently, the exercise duration increased by 5–10% weekly, reaching 60 min by the end of the intervention. Exercise intensity was prescribed and monitored based on participants’ heart rate (HR), targeting 50 to 60% of their maximum HR (HRmax = 207 − 0.7 × age) [18]. Since many HD patients use beta-blockers, the modified Borg’s RPE scale [19] was also employed to subjectively control the exercise intensity. The range of the modified RPE scale is 0 to 10, where 0 represents no exertion and 10 represents maximal effort. Participants were asked to cycle at a self-selected pace corresponding to an RPE of 3–4, corresponding to “mild” to “quite hard” effort.

For resistance training, the initial weights for knee extension, hip abduction, and flexion exercises were determined using a three-repetition maximum (3-RM) test [5]. Participants performed 2–3 sets of 10 repetitions at approximately 60% of 3-RM using ankle weights. When participants could complete three sets with the correct technique, the weight was increased. Recovery periods were 1 min between sets and 3 min between exercises. Throughout the training session, exercise intensity and vital signs (blood pressure, heart rate, and oxygen saturation (SpO_2_)) were monitored every 5 min. After exercising, lower-limb stretching exercises were performed, targeting the hamstrings, hip adductors, hip abductors, tibialis anterior, gastrocnemius, and soleus muscles. The exercise regimen was discontinued if participants experienced any of the following indications or symptoms: (i) chest pain, (ii) arrhythmias, (iii) dyspnea, (iv) nausea, (v) muscle pain or cramps, (vi) episodes of hypotension or hypertension, or (vii) a high RPE score on the modified Borg’s scale. The C-PLA group continued their standard HD sessions without intradialytic exercise.

### 2.5. Blood Sampling and Assays

Patients were instructed to fast overnight for at least 8 h and refrain from exercising for 24 h before blood sample collection. Resting blood samples (~10 mL) were obtained from the antecubital vein both before (one day prior to the first training session) and after training (one day after the final training session). These samples were collected at the same time of day, between 07:00 and 07:30 AM, to minimize circadian fluctuations. Blood samples were divided into three tubes: one containing K2EDTA (3.5 mL), one containing lithium heparin (4 mL), and one without anticoagulant (2.5 mL). Samples were placed in an ice bath and immediately centrifuged at 2500 rpm and 4 °C for 10 min. Aliquots of the resulting plasma were stored at −80 °C until analysis. K2EDTA tubes were used to determine MDA, GSH, THIOL, and FRAP. Plasma levels of TBIL, UA, Gamma-GT, ASAT, ALAT, CK, LDH, and CRP were assessed using lithium heparin tubes. To reduce inter-assay variance, all samples were analyzed in the same assay run.

#### 2.5.1. Determination of Lipid Peroxidation (MDA)

Following the method described by Wong et al. [20], a 0.5 mL plasma sample was mixed with 0.1 mL of tris-HCL buffer (pH 7.2) and incubated at 95 °C in a water bath. After incubation, 0.5 mL of the mixture was combined with 9 mL of distilled water and 2 mL of 0.6% thiobarbituric acid and then heated for 30 min in a boiling water bath. After cooling, 5 mL of n-butanol was added, and the mixture was vigorously stirred. The n-butanol layer was then separated by centrifugation at 3000 rpm for 10 min, and the malonic MDA content was quantified using a spectrophotometer at 532 nm (Libra S21, Biochrom, Holliston, MA, USA).

#### 2.5.2. Measurement of FRAP Levels

The determination of FRAP was conducted using the method of Benzie and Strain with a slight modification [21].

- Principle: This reaction involves the reduction of the ferric tripyridyltriazine complex (TPTZ-Fe^3+^) to the ferrous form (Fe^2+^) in an acidic medium by antioxidants present in the sample.
- Required reagents include: Acetate buffer (pH 3.6), which is a mixture of two solutions: 46.3 mL of solution A and 3.7 mL of solution B. Solution A consists of 0.2 M acetic acid (11.55 mL in 1 L of distilled water), with a molar mass of 60.052 g/mol. Solution B consists of 0.2 M sodium acetate (16.4 g sodium acetate in 1 L of distilled water). The reactive mixture (1:1) comprises 20 mM ferric chloride [FeCl_3_•6H_2_O] and 10 mM tripyridyltriazine in 40 mM HCl. Ferric sulfate (FeSO_4_) was used to create the calibration curve [1 − 0.1 mmol/L]. The method was calibrated using a standard solution of hydrated ferrous sulfate FeSO_4_•5H_2_O with concentrations ranging from 0 to 1.5 mM. The reducing capacity of the sample was expressed in equivalents of ferrous ions in plasma (μmol Fe^2+^/L).
- Calculation: The FRAP concentrations were calculated using the formula [FRAP] (μmol/L) = DO − b/a × 1000, where DO is the optical density, ‘a’ is the slope of the Fe^2+^O_4_ calibration curve (mmol/L), ‘b’ is the y-intercept, and 1000 is the conversion factor from mmol to μmol.

#### 2.5.3. Measurement of GSH Levels

Determination of GSH levels was carried out using a colorimetric method with Ellman’s reagent: 5,5′-dithiobis-(2-nitrobenzoic acid) (DTNB) [22]. The principle involves the reaction of GSH with DTNB, releasing thionitrobenzoic acid (TNB), which has an absorbance at 412 nm. The necessary reagents include a 4% aqueous solution of sulfosalicylic acid, and a Tris-EDTA buffer solution (0.25 M Tris base + 0.02 M EDTA) composed of two solutions: Tris base (0.5 M) prepared by weighing 3 g in 50 mL distilled water, and EDTA (0.04 M) prepared by weighing 0.58 g in 50 mL distilled water. These solutions are mixed (*V*/*V*) to obtain the Tris-EDTA buffer, with the pH adjusted to 8.2. A freshly prepared 10 mM DTNB solution was made by dissolving DTNB in an adequate volume of methanol, and the bottle was kept away from light by wrapping it in aluminum foil. GSH was used to create the calibration curve [1 − 0.03 mmol/L]. GSH levels were determined using Jollow et al.’s method [23]. The calculation, based on a standard curve prepared with GSH, expresses the result in nanomoles per milliliter of plasma, then is adjusted to protein levels (nmol GSH/mg protein).

The formula used is [GSH] (nmol/mg) = (DO − b/a × FD × 1000)/[proteins mg/mL], where ‘DO’ is the optical density, ‘a’ is the slope of the GSH calibration curve (μmol/mL), ‘b’ is the y-intercept, FD is the dilution factor, and 1000 is the conversion factor from μmol to nmol.

#### 2.5.4. Determination of THIOL Levels

Following Hu’s methodology [24], THIOL levels were evaluated using a spectrophotometric technique (Libra S21, Biochrom, Holliston, MA, USA). A mixture of 50 µL of plasma and 1000 µL of Tris-EDTA buffer (0.25 M, 20 mM, pH 8.2) was prepared, with the initial absorbance recorded at 412 nm. Subsequently, 20 µL of DTNB (10 mM) was added to the solution, which was then protected from light. After 10 min, the absorbance was measured again. The difference between the initial and final absorbance values was used to calculate the THIOL concentration, adjusting for the standard range coefficient and plasma protein concentration.

#### 2.5.5. Routine Biochemistry Parameters

The Cobas 6000^®^ (Module c501, Roche Diagnostics, Mannheim, Germany) automated biochemistry analyzer was used to measure TBIL, UA, ASAT, ALAT, CK, LDH, Gamma-GT, and CRP. TBIL was tested using the Diazo reaction [25]. An enzymatic technique was employed to determine UA at 550 nm [26]. The concentrations of ASAT, ALAT, and CK levels were assessed using the N-acetyl-L-cysteine enzymatic technique [27]. According to the IFCC, LDH concentration was measured using the UV test, and Gamma-GT was measured using the colorimetric enzymatic assay (L-γ-glutamyl carboxy-3-nitro-4-anilide as the substrate) [28]. CRP was measured using the immunoturbidimetric technique [29].

### 2.6. Statistical Analysis

The data are presented as mean ± standard deviation (SD) in the tables and figures. The normality of the distribution was assessed and confirmed using the Shapiro–Wilk test prior to statistical analysis. Differences between group characteristics were examined using one-way ANOVA. Additionally, two-way repeated measures ANOVA [Groups (EX-MEL vs. EX-PLA vs. C-PLA) × Time (pre-training vs. post-training)] was used to analyze the data. Practical significance of the ANOVA was evaluated using partial eta-squared (ηp2). When necessary, the Bonferroni post-hoc test was used for pairwise comparisons. Effect sizes were calculated using Cohen’s d method and interpreted as small (<0.3), medium (<0.5), or large (>0.8) [30]. Statistical significance was set at *p* < 0.05. All statistical analyses were performed using Statistica 10 software (StatSoft, Maisons-Alfort, France).

## 3. Results

Statistical analysis of MDA, FRAP, GSH, and THIOL showed significant effects of time (F_(1,31)_ = 23.87, *p* < 0.001, ηp2 = 0.72; F_(1,31)_ = 25.04, *p* < 0.001, ηp2 = 0.73; F_(1,31)_ = 37.98, *p* < 0.001, ηp2 = 0.80; and F_(1,31)_ = 26.04, *p* < 0.001, ηp2 = 0.74, respectively). Moreover, significant interactions (Time × Groups) were observed for THIOL and CRP (F_(2,30)_ = 4.89, *p* < 0.05, ηp2 = 0.35 and F_(2,30)_ = 6.70, *p* < 0.01, ηp2 = 0.42, respectively). However, no significant variations in TBIL, UA, CK, LDH, ASAT, ALAT, or Gamma-GT were observed pre- and post-training in all groups (Table 2).

**Figure 2:**
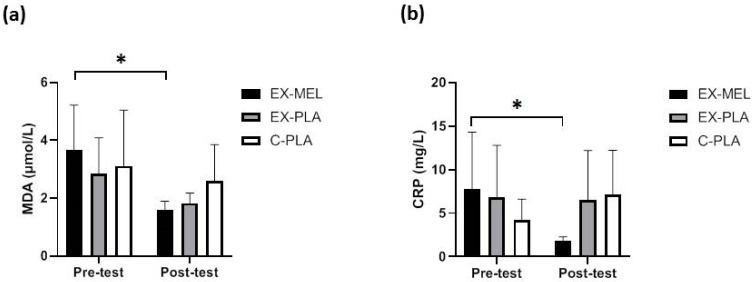
Effect of intradialytic concurrent training and melatonin supplementation on lipid peroxidation and inflammation in hemodialysis patients. Malondialdehyde (MDA) (**a**); C-reactive protein (CRP) (**b**). EX-MEL: Exercise-Melatonin; EX-PLA: Exercise-Placebo; C-PLA: Control-Placebo; Pre- and Post-test correspond to data collection before and after 12 weeks of intervention. * *p* < 0.05: significant difference between pre- and post-test.

**Figure 3:**
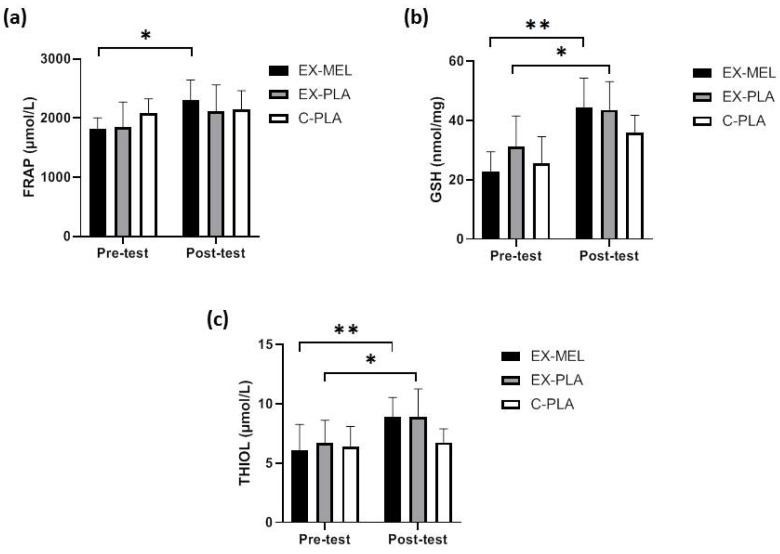
Impact of concurrent intradialytic training and melatonin supplementation on biomarkers of the antioxidant system in hemodialysis patients. Ferric-reducing antioxidant power (FRAP) (**a**); reduced glutathione (GSH) (**b**); total thiol (THIOL) (**c**); EX-MEL: Exercise-Melatonin; EX-PLA: Exercise-Placebo; C-PLA: Control-Placebo; Pre- and Post-test correspond to data collection before and after 12 weeks of intervention. * *p* < 0.05, ** *p* < 0.01: significant difference between pre- and post-test.

EX-MEL showed significant improvements in lipid peroxidation (MDA) (*p* < 0.05, d = 2.24) (Figure 2a), total antioxidant capacity (FRAP) (*p* < 0.05, d = 1.83) (Figure 3a), and CRP (*p* < 0.05, d = 1.70) (Figure 2b) pre- and post-training.

EX-MEL and EX-PLA showed significant increases in GSH (*p* < 0.01, d = 2.57 and *p* < 0.05, d = 1.24, respectively) (Figure 3b) and THIOL (*p* < 0.01, d = 1.46; *p* < 0.05, d = 1.01, respectively) (Figure 3c) pre- and post-training. Nevertheless, no significant differences were noted between EX-MEL and EX-PLA.

## 4. Discussion

This study investigated the combined effects of ICT and MEL supplementation on oxidative stress, inflammation, and cellular damage in HD patients. The results showed that both the EX-MEL and EX-PLA groups exhibited notable increases in GSH and THIOL levels, while the EX-MEL group showed significant improvements in MDA, FRAP, and CRP levels pre- and post-training. The potential benefits of this combined intervention highlight significant improvements in markers of oxidative stress and inflammation.

ICT showed positive effects on oxidative stress markers, consistent with prior studies on exercise benefits in HD patients [31,32]. In the present study, both the EX-MEL and EX-PLA groups demonstrated significant increases in GSH and THIOL levels, indicating enhanced antioxidant defenses. These findings support the idea that exercise during dialysis sessions improves the oxidant–antioxidant balance [6,33]. Regular exercise enhances antioxidant enzyme activity and reduces oxidative stress in various populations, involving those with chronic kidney disease (CKD) undergoing HD [34]. The mechanisms by which ICT enhances antioxidant defenses include the upregulation of key antioxidant enzymes like superoxide dismutase (SOD), catalase (CAT), and glutathione peroxidase (GPx), which detoxify ROS [35]. Exercise stimulates GSH synthesis, enhancing the cells’ capacity to neutralize free radicals and regenerate other antioxidants [36], and improves mitochondrial function, reducing electron leakage and ROS formation, thereby lowering oxidative stress [37]. Additionally, exercise activates the Nrf2 pathway, promoting the expression of antioxidant and cytoprotective genes that maintain cellular redox homeostasis [38] and decreasing the levels of pro-oxidant enzymes like NADPH oxidase and xanthine oxidase, reducing ROS production [39].

MEL’s antioxidant and anti-inflammatory properties are well-established, but its specific effects on HD patients have been less explored [5,11,12]. The EX-MEL group’s significant improvements in MDA, FRAP, and CRP levels highlight MEL’s potential to enhance the benefits of exercise by further reducing oxidative stress and inflammation [11,12]. This synergy could be due to MEL’s ability to scavenge ROS and upregulate antioxidant enzymes, thereby amplifying the exercise-induced protective effects [40]. MEL directly neutralizes ROS such as hydroxyl radicals and superoxide anions, reducing oxidative stress [41]. Moreover, MEL indirectly enhances the activities of antioxidant enzymes such as SOD and GPx [41]. In HD patients, MEL supplementation reduces oxidative stress markers, including MDA and advanced oxidation protein products, while increasing biomarkers of the antioxidant system, such as CAT and THIOL [12].

MEL also inhibits pro-inflammatory cytokines like tumor necrosis factor-alpha (TNF-α), interleukin-6 (IL-6), and interleukin-1 beta (IL-1β), and suppresses nuclear factor kappa B (NF-κB), a key transcription factor involved in inflammation [42,43]. Importantly, no side effects of MEL supplementation were observed in the present study, consistent with the findings of Marzougui et al. [5] and Koch et al. [16]. The improvements in MDA, FRAP, and CRP levels in the EX-MEL group underscore MEL’s potential to enhance exercise-induced benefits, offering a promising adjunct therapy for HD patients [5,12].

TBIL and UA levels pre- and post-training did not change significantly across all groups. This outcome highlights the safety and metabolic stability of the combined intervention of ICT and MEL supplementation in HD patients. Bilirubin, a byproduct of hemoglobin breakdown processed in the liver, serves as a marker for liver function and hemolysis [44]. Elevated levels can indicate liver dysfunction or hemolysis, while low levels may signal reduced red blood cell turnover [45]. In this study, the unchanged TBIL levels post-intervention suggest that neither the exercise regimen nor MEL supplementation imposed additional stress on the liver, which is crucial for HD patients with compromised liver function due to CKD [44].

Similarly, UA, a product of purine metabolism, is often elevated in CKD patients due to reduced renal clearance [46]. High UA levels can contribute to gout and cardiovascular complications [47,48]. The absence of significant changes in UA levels post-intervention indicates that the interventions did not adversely impact purine metabolism. Additionally, it suggests that they did not exacerbate UA accumulation [46,47]. These findings underscore the metabolic stability of the combined exercise and MEL supplementation regimen in HD patients, supporting its safety and potential clinical benefits.

Interestingly, no significant changes in biomarkers of muscle and liver damage (CK, LDH, ASAT, ALAT, and Gamma-GT) were found across all groups pre- and post-intervention. This suggests that the concurrent training program, whether combined with MEL supplementation or not, did not exacerbate muscle or liver injuries in HD patients. This finding is crucial, as it indicates that the prescribed exercise regimen is safe and does not lead to additional cellular damage in these patients.

CK and LDH are commonly used biomarkers for muscle damage, while ASAT, ALAT, and Gamma-GT are indicators of liver damage [49]. Elevated levels of these enzymes can signal tissue damage or stress [50]. The lack of significant changes in these biomarkers suggests that the exercise intensity and duration were well-tolerated by the participants. This aligns with existing research showing that appropriately monitored exercise does not necessarily cause harm to HD patients and can be a safe part of their therapeutic regimen [5,12].

Based on previous studies showing enhanced benefits in oxidative stress [12] and inflammation [11] from a single dose of MEL before acute exercise in HD patients, we initially hypothesized that a daily dose of MEL over three months, combined with ICT, would yield synergistic effects rather than merely additive ones. Although our study did not reveal statistically significant improvements in the EX-MEL group compared to EX-PLA or C-PLA, we consider the effects to be synergistic due to distinct improvements observed only in the EX-MEL group. Specifically, reductions in lipid peroxidation (i.e., MDA), enhanced antioxidant capacity (i.e., FRAP), and decreased inflammation (i.e., CRP) were uniquely observed in the group receiving both MEL and exercise. These changes imply that MEL, in conjunction with ICT, functions complementarily to attenuate oxidative and inflammatory responses, rather than merely adding to the benefits of exercise alone.

While exercise is beneficial, it may also exacerbate oxidative stress and inflammatory responses, particularly in this population with elevated baseline levels of these markers [51]. MEL may mitigate these adverse effects, providing a protective role against potential exercise-induced oxidative stress [42]. This interaction between MEL and exercise supports the hypothesis of a synergistic effect rather than an additive one.

Thus, while the effects were not statistically “greater,” the qualitative differences in biomarker responses between the EX-MEL and EX-PLA groups support the idea of a synergistic interaction.

The outcomes of the current study have important clinical implications for the management of oxidative stress and inflammation in HD patients, which provides a strong rationale for integrating these interventions into routine HD care. It demonstrates that a combination of ICT and MEL supplementation can yield significant health benefits without causing muscle or liver damage. Future studies should explore the long-term effects of this combined approach and investigate the underlying mechanisms driving these benefits.

While the results are promising, the study has some limitations. The sample size, although calculated to ensure sufficient power, is relatively small, and the duration of the intervention was limited to 12 weeks. Additionally, this analysis did not consider potential differences in patients’ sex. Larger sample size and longer-term studies are needed to confirm these findings and evaluate the sustainability of the observed benefits.

## 5. Conclusions

This study provides compelling evidence that combining ICT with MEL supplementation can significantly reduce oxidative stress and inflammation in HD patients without causing muscle or liver damage. These findings suggest a novel therapeutic strategy that could improve health outcomes and quality of life among individuals undergoing HD. Further research is warranted to validate these results and explore the broader implications of this combined intervention.

## Figures and Tables

**Table 1:** Baseline Characteristics by Group Allocation (n = 32).

Characteristic	EX-MEL (n = 11)	EX-PLA (n = 11)	C-PLA (n = 10)	*p*-Value
Sex (male/female)	8/3	7/4	4/6	
Age (years)	49.27 ± 10.23	49.00 ± 12.51	41.00 ± 9.68	0.88
Height (cm)	166.72 ± 7.01	166.00 ± 9.48	162.70 ± 10.62	0.79
Dry weight (kg)	61.95 ± 6.22	63.44 ± 6.66	65.20 ± 14.36	0.83
BMI (kg/m^2^)	22.37 ± 2.79	23.25 ± 3.99	24.54 ± 3.99	0.60
Hemodialysis (months)	105.68 ± 18.78	115.79 ± 28.48	124.50 ± 24.37	0.67
Urea (mmol/L)	22.21 ± 5.10	21.94 ± 4.41	27.99 ± 6.03	0.29
Creatinine (µmol/L)	944.09 ± 158.75	976.90 ± 205.99	992.06 ± 157.04	0.47
Primary disease (n)				
Glomerulonephritis	4	3	2	
Chronic interstitial nephritis	3	4	3	
Family nephropathy	2	3	2	
Uncertain	2	1	3	

BMI: body mass index.

**Table 2:** Effects of combined intradialytic training and melatonin supplementation on cellular damage markers.

Variables	EX-MEL (n = 11)		EX-PLA (n = 11)		C-PLA (n = 10)		ANOVA		
	Pre	Post	Pre	Post	Pre	Post	Groups Effect F_(2,30)_ (*p*; ηp2)	Time Effect F_(1,31)_ (*p*; ηp2)	Interaction F_(2,30)_ (*p*; ηp2)
CK (U/L)	73.90 ± 28.66	80.36 ± 31.63	86.36 ± 36.15	88.90 ± 39.41	69.1 ± 32.96	88.90 ± 36.66	0.52 (*p* = 0.60; 0.05)	2.00 (*p* = 0.19; 0.18)	1.34 (*p* = 0.28; 0.13)
LDH (UI/L)	167.90 ± 25.52	162.09 ± 22.63	186.45 ± 41.12	181.63 ± 44.02	183.90 ± 44.76	187.10 ± 46.04	1.05 (*p* = 0.37; 0.10)	0.18 (*p* = 0.67; 0.02)	0.30 (*p* = 0.73; 0.03)
ASAT (U/L)	14.15 ± 6.76	11.79 ± 4.38	11.90 ± 4.91	11.78 ± 6.81	10.80 ± 4.06	13.89 ± 5.78	0.02 (*p* = 0.98; 0.002)	0.001 (*p* = 0.96; 0.0002)	4.16 (*p* = 0.03; 0.31)
ALAT (U/L)	11.75 ± 6.39	9.46 ± 3.86	8.66 ± 5.69	8.47 ± 4.03	4.46 ± 2.48	10.46 ± 4.08	1.20 (*p* = 0.32; 0.11)	0.52 (*p* = 0.48; 0.05)	9.13 (*p* < 0.01; 0.50)
Gamma-GT (IU/L)	28.27 ± 15.57	24.09 ± 15.67	25.63 ± 15.53	20.27 ± 10.86	22.60 ± 9.14	18.70 ± 4.66	0.34 (*p* = 0.70; 0.03)	9.79 (*p* < 0.01; 0.52)	0.08 (*p* = 0.91; 0.009)
TBIL (µmol/L)	4.90 ± 0.70	5.54 ± 1.57	5.09 ± 1.70	4.90 ± 1.30	4.10 ± 1.10	4.90 ± 1.37	0.88 (*p* = 0.43; 0.08)	3.31 (*p* = 0.10; 0.26)	1.75 (*p* = 0.20; 0.16)
UA (µmol/L)	370.45 ± 72.81	319.36 ± 85.16	391.09 ± 84.31	376.27 ± 78.22	372.30 ± 62.73	349.40 ± 41.16	1.23 (*p* = 0.31; 0.12)	3.68 (*p* = 0.08; 0.29)	0.66 (*p* = 0.52; 0.06)

EX-MEL: Exercise-Melatonin; EX-PLA: Exercise-Placebo; C-PLA: Control-Placebo; Pre and Post correspond to data collection before and after 12 weeks of intervention. CK: creatine kinase; LDH: lactate dehydrogenase; ASAT: aspartate aminotransferase; ALAT: alanine aminotransferase; Gamma-GT: gamma-glutamyl transferase; TBIL: total bilirubin; UA: uric acid.

## Data Availability

The data presented in this study are available on request from the corresponding author. The data are not publicly available due to confidentiality.

## Supplementary Materials

The following supporting information can be downloaded at: https://www.mdpi.com/article/10.3390/antiox13111290/s1, Table S1: CONSORT checklist.
